# A study on blood product usage and wastage at the public hospital, Guyana

**DOI:** 10.1186/s13104-016-2112-5

**Published:** 2016-06-13

**Authors:** Rajini Kurup, Audrey Anderson, Cecil Boston, Lynn Burns, Marian George, Marana Frank

**Affiliations:** Faculty of Health Sciences, University of Guyana, Georgetown, Guyana, South America

**Keywords:** Blood usage, Blood wastage, Transfusion

## Abstract

**Background:**

Blood is a valuable resource and blood wastage in a low socio economic country could impose a very serious impact on healthcare. This study therefore analyzes the usage and wastage of blood and blood products at the Georgetown Public Hospital Cooperation (GPHC), Guyana.

**Methods:**

A retrospective study was conducted on the data retrieved from laboratory blood banking information system on usage and wastage of blood products during the years 2012–2014 at the public hospital. The data were analyzed in MS Excel and SPSS 20.0.

**Results:**

A total of 16,426 units of blood were issued from National Blood Transfusion Services. During the study period the most frequently requested blood component was packed cells followed by fresh frozen plasma (FFP), platelet, cryoprecipitate (CRYO) and whole blood respectively. Data indicated that 4167 units (25 %) of blood were wasted due to various reasons at GPHC.

**Conclusions:**

There is a need for intervention through raising awareness among medical staff in reducing blood wastage.

## Background

Blood and its components are very significant for human life and therefore blood transfusion can be a life-saving intervention. There are multiple factors that contribute to shortfall in provision of blood including deficient donor recruitment, poor stock management and transportation. The demand for blood surpasses the blood supply in many countries. World Health Organization (WHO) data indicated that 87.5 % of developing countries collect less than half of the blood needed to meet the transfusion requirements of their populations [1]. Studies on developing countries reported that most of the limited blood supplies are used for complications of pregnancy and childbirth, trauma and severe anemia in childhood [2–4].

Many factors lead to wastage of blood products like broken bag, broken seal, expired units, returned after 30 min, clotted blood or miscellaneous reasons which is most importantly due to lack of proper knowledge and awareness. According to the “30-minute rule” and guidelines for blood transfusion in the UK recommend that if RBC units are out of controlled temperature storage for more than 30 min, they should not be put back into storage for reissue [5]. The justification for this rule is that once RBC units are out of controlled temperature storage, the component warms up, and the risk of bacterial proliferation increases with time [6, 7].

Ideally in a proper setting, outdating and wastage of blood and blood products would never occur. Due to the inherent need to have blood stocks at all times and also often unpredictable demands on the inventory, a very limited and inevitable outdating of components in blood bank is accepted [8]. Studies claim that through target interventions and adherence to strict guidelines, a significant reduction in the wastage of blood components could be achieved and maintained [9–11]. Globally only 106 countries have national guidelines on the appropriate clinical use of blood and blood products [12].

In Guyana, GPHC is the country’s referral hospital which always has high demand for blood for transfusion and National Blood Transfusion Services, Guyana is responsible for ensuring and providing adequate supply of blood components. Therefore, this is a first attempt to analyze the data on blood utilization in GPHC and would provide a helpful guideline for optimum use of blood and blood products.

## Methods

This study is a quantitative retrospective cross sectional descriptive study, data relating to collection and usage of blood and its component from July 2012 to December 2014 at GPHC laboratory. Data on blood and its component usage and wastage were collected from GPHC laboratory blood banking information system. However, due to the unavailability of data for the first half of 2012, the researchers were unable to acquire data from Jan 1st to July 22nd 2012. The data were classified according to blood usage and wastage, so as to prevent any data mix-up. Data were first entered in MS Excel and later analyzed using SPSS 20.0.

### Hospital system on blood transfusion

Blood collection, processing and screening blood from donors is done at the Blood bank of National Blood Transfusion Services (NBTS). It is a centralized blood center which collects blood directly or through blood drives from mostly voluntary blood donors. NBTS also has blood facilities at New Amsterdam, West Demerara, Suddie and Linden Hospitals along with mobile units for blood camps. Central laboratory at GPHC is responsible to collect the blood from blood bank as per the request/protocol at the hospital. A minimum inventory is required at the hospital comprising 62, 60, 40 and 26 units of packed cells, FFP, CRYO and platelets respectively. However, demand is rarely if ever met requiring daily submission of requests for blood from the blood center.

All data collected for blood requests and wastage was processed and categorized as follows

1. Comparative utilization of components.
2. Reasons for wastage (discard) of blood components.

There were no records available on the reasons for usage (issue) of blood components at GPHC.

However, blood wastage were recorded for a number of reasons: time expiry (the expiry date on the unit has passed), broken seal, broken bag, broken cold chain, clotted blood, blood returned after 30 min.

### Ethical considerations

Ethical approval for the study was obtained from Institution Review Board, Ministry of Health, Guyana and Director of GPHC Laboratory before proceeding with the research.

## Results

### Blood distributions

**Fig. 1:**
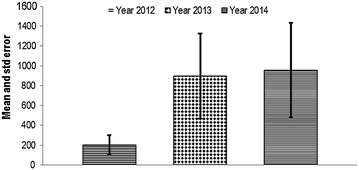
Mean value and standard error of blood units used during the study period

A total of 16, 426 units of blood were collected at GPHC from blood bank during July 2012 to December 2014. Each year recorded a blood unit collection of 1616 (9.8 %), 7159 (43.6 %), 7651 (46.6 %) respectively. Mean (average) blood unit with standard error each year is shown in the Fig. 1. Mean blood unit (± SE) collected during the study period were 202.0 ± 275.5 (95 % CI 188.6–215.4), 894.9 ± 1216.4 (95 % CI 866.7–923.1) and 956.4 ± 1348.5 (95 % CI 926.2–986.6) for the years 2012, 2013 and 2014 respectively.

### Monthly report of blood distribution

**Fig. 2:**
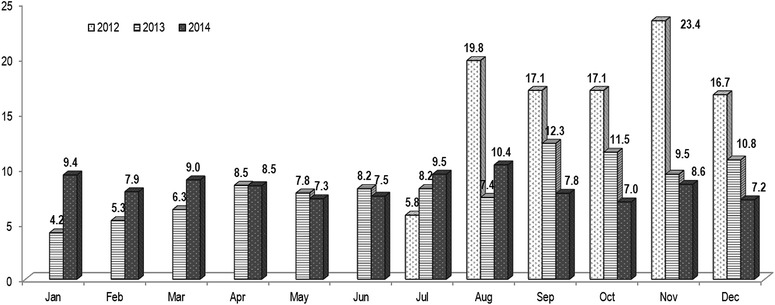
The monthly distribution of blood units (%) during the study period

Data for the year 2012 was recorded from the month of July due to the lack of computerized system on place. Monthly break down of blood units collected each year is shown in Fig. 2. The highest collection of blood units were during the year 2012 and was during the month of November (23.4 %). For the year 2013 September month had highest (12.3 %) blood unit collection whereas in January had least (4.2 %) blood unit’s collected. For the year 2014, August had the most amount of blood collection (10.4 %) followed by July and January with 727 and 722 units respectively. Therefore, during the years 2013 and 2014, GPHC collected an average of approximately 600 units of blood on a monthly basis and most blood collections were recorded during the last half of the year. Overall there is no particular trend in collection for blood in a monthly basis.

### Blood group collected during the study period

**Fig. 3:**
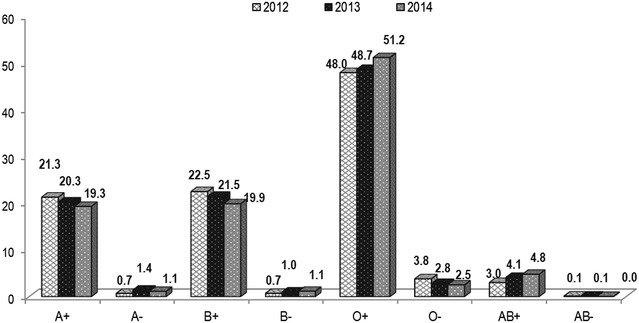
Percentage of blood group distribution during the study period

Figure 3 demonstrates the distribution of blood group during the study period. It was observed that O+ blood type was the most frequently collected blood type with 775 (48.0 %), 3484 (48.7 %), 3919 (51.2 %) during the years 2012, 2013 and 2014 respectively. The second most collected blood group at the hospital was B+ with 363 units (22.5 %) for the year 2012, 1542 units (21.5 %) for 2013 and 1522 units (19.9 %) for 2014 and A+ had a recorded 345 (21.3 %) for 2012, 1450 (20.3 %) for 2013 and 1479 (19.3 %) blood units for the year 2014. The least collected blood group at GPHC was the negative groups with AB− being the least blood group collected.

### Blood components collected during the study period

Table 1 demonstrates the distribution of blood component collected during the study period 2012–2014. The most collected blood component was packed cells (70.1 %), followed by platelet (13.4 %), fresh frozen plasma (12.8 %), cryoprecipitate (3.0 %) and whole blood (0.6 %) respectively. For the year 2014, whole blood was not collected by the hospital at no point.

**Table 1:** Total units of blood component collected during the study period

Blood components collected	2012	2013	2014
Cryoprecipitate	45 (2.8 %)	279 (3.9 %)	165 (2.2 %)
Whole blood	42 (2.6 %)	62 (0.9 %)	0
Packed cell	1338 (82.8 %)	4952 (69.2 %)	5225 (68.3 %)
Fresh frozen plasma	54 (3.3 %)	867 (12.1 %)	1189 (15.5 %)
Platelet	137 (8.5 %)	999 (14 %)	1072 (14 %)

### Blood wastage during the study period

**Fig. 4:**
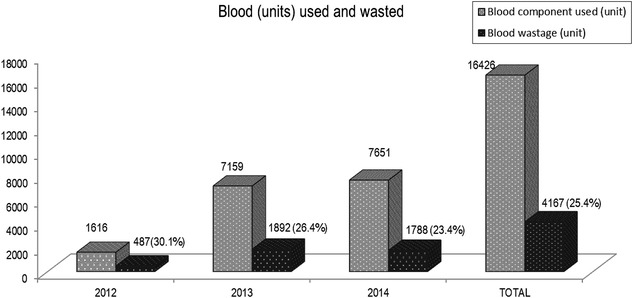
Total blood units used and wasted during the study period

Blood wastage at GPHC during the study period is demonstrated in Fig. 4. An overall of 4167 (25.4 %) blood units were wasted during the study period. Blood units wasted during the year 2012, 2013 and 2014 were recorded as 487 (30.1 %), 892 (26.4 %) and 1788 (23.4 %) respectively. Most frequently discarded blood group was O+ and blood component was platelet during the study period.

### Reasons for blood wastage

Table 2 demonstrates the various causes of blood wastage during the study period. The major reasons for blood wastage include expired blood unit, broken seal, broken cold chain, broken bag, returned after 30 min, clotted blood and the least being component with red cells. Blood wastage during the years 2012, 2013 and 2014 were recorded as 487 (11.7 %), 1892 (45.5 %) and 1788 (42.9 %) units respectively.

**Table 2:** Reasons for blood wastage in GPHC

Reasons for blood wastage	2012	2013	2014
Broken bag	6 (1.2)	26 (1.4)	30 (1.7)
Broken seal	3 (0.6)	7 (0.4)	99 (5.5)
Broken cold chain	2 (0.4)	3 (0.2)	63 (3.5)
Clotted blood	1 (0.2)	17 (0.9)	12 (0.7)
Component with red cells	2 (0.4)	2 (0.1)	3 (0.2)
Expired unit	471 (96.6)	1826 (96.5)	1568 (87.7)
Return after 30 min	2 (0.4)	11 (0.6)	13 (0.7)
n (%)			

## Discussion

This study attempts to analyze the blood usage and wastage at GPHC during the period 2012–2014. A close view on the distribution of blood would help to focus on the areas of frequent wastage and to understand- the main cause/causes of blood wastage. This would further help to design intervention programs or measures to prevent such wastage and also to increase awareness of the wast-age issue throughout the hospital in general. Wastage of blood components will continue to be an issue at all hospitals therefore inexpensive and easy interventions such as educational outreach, print and digital messaging, and improved transportation and component identification modalities can have a prompt and dramatic impact on reducing blood wastage with regard to both cost and resource savings [13, 14]. Cryoprecipitate and FFP have a shelf life of 24 h once prepared and it has been noted in the study that sometimes doctors order them and later change their mind due to different reasons. Because blood products have a limited half-life, accurate strategies should have been enforced for blood reserves in order to prevent loss and reduce wastes as much as possible [15].

On reviewing with blood bank staff, it was noted that the major reason for wastage of different components might be due to how it was handled after collection from the laboratory. It was also recommended that there is always larger shortage of blood and a need of awareness is important among laboratory staffs, nurses and physicians on handling blood products.

Most collected blood group during the study period was O+ when compared to the other groups followed by B+ and A+ blood groups. O+ being the most common blood type therefore more patients needs it. Moreover, O+ is the universal donor for RBCs and compatible with other blood types and can be transfused during emergency. Annually more than 120,000 O+ units of blood, platelets and plasma are required to meet the needs of the hospitals [16]. The least blood group collected during the study period was AB− because fewer patients were AB−. There is no study available that supports the distribution of ABO blood groups among general population or blood donors in Guyana.

Of the five blood components collected at GPHC, packed cells were most frequently collected followed by fresh frozen plasma, platelet, cryoprecipitate and whole blood respectively. Packed cells were the most frequent blood component in another study in comparison to the study by Singhall which found whole blood being the frequently collected blood component [17, 18]. Whole blood was the least collected blood component and this could be because whole blood is not frequently administered to patients unless more than 75 % of blood was lost.

GPHC follows a guideline which determines when blood or blood component should be discarded and these criteria’s are broken bag, broken seal, broken cold seal, expiration and contamination. Major cause of blood wastage in this study was due to expiration followed by broken seals, broken cold chain, broken bag and the least being component with red cells. Similar findings were seen in other studies, expired blood units being major cause of wastage [14, 17] in contrast to study by Kora, which showed contamination being the major reason of blood wastage [19].

Most frequently discarded blood group was O+ in the study and the most frequently discarded blood component was platelets. Because platelets have a shorter shelf life of 5 days, it should be used within 4 h after pooling. Platelet is the most commonly discarded component in a similar other studies [20].

This study being retrospective one, only information’s available in the records was collected for the study. Due to this limitation it was not possible to determine the rate of wastage by in-hospital location.

## Conclusions

The study showed a 25 % of blood wasted at the GPHC, the most being O+ blood group and platelets. Wastage of blood in hospitals is a universal issue and should be addressed with easy and inexpensive interventions that can reduce wastage of blood and blood components. Programs to encourage blood donations should be increased so as to accommodate the increased blood usage. Finally, the study hopes to draw the attention of the blood wastage in GPHC and to decrease the amount of blood wastage, as blood is an important and rare resource.

## Abbreviations

GPHC: Georgetown Public Hospital Cooperation
WHO: World Health Organization
